# Describing what the body plays: the BODYPERC checklist for reporting body percussion interventions, a method-neutral extension of TIDieR and CERT

**DOI:** 10.3389/fpsyg.2026.2004833

**Published:** 2026-10-22

**Authors:** Francisco Javier Romero-Naranjo, Maria Luisamercedes Gonzalez-de-Benatuil

**Affiliations:** Department of Innovation and Educational Training, University of Alicante, Alicante, Spain

**Keywords:** active control group, body percussion, CERT, dance and music interventions, dual-task, intervention reporting, TIDieR, treatment fidelity

## Abstract

Body percussion, the production of rhythm by clapping, snapping, patting and stamping on one’s own body, is increasingly used as an intervention in schools, rehabilitation settings and community programmes for older adults, often within dance- and music-based approaches. Evidence synthesis is limited less by a shortage of studies than by how interventions are described. Two programmes labelled “body percussion” can differ in the body surfaces struck, the tempo, the organisation of the limbs, the concurrent cognitive task, the social formation and the dose, and research on bimanual coordination, sensorimotor synchronisation, dual-tasking and interpersonal synchrony suggests that each of these features can change what participants do and, plausibly, what they gain. Reports usually specify only some. TIDieR describes any intervention but does not prompt its rhythmic or motor content; CERT specifies exercise dose but not musical structure or cognitive load; and reporting guidance for music-based interventions addresses the music but not the body as instrument. This conceptual analysis proposes BODYPERC, a method-neutral checklist of 33 items in 11 domains for reporting body percussion interventions and their comparators. Items were derived by mapping existing guidelines and the implementation-fidelity literature onto five features that distinguish body percussion as an intervention class: the coincidence of effector and instrument, rhythmic structure as a dose parameter, deliberately manipulated cognitive load, interpersonal synchrony as a social ingredient, and repertoire drawn from living cultural traditions. The checklist requires interventions to be described by their features rather than by a method’s name, specifies the equivalences a comparison condition must meet before it can be called an active control, and adds process measures (timing precision, interpersonal synchrony and dual-task cost) that allow delivery to be verified from audio recordings or wearable sensors. Applied to three purposively selected reports, it identified features that none reported, including tempo, auditory context, fidelity monitoring and adverse events; whether these gaps are typical of the literature requires a structured audit, whose protocol is provided. The main limitation is that BODYPERC derives from conceptual analysis, not expert consensus; an audit, a Delphi study and a reliability study are required before it can become a standard.

## 1 Introduction

Body percussion is the production of rhythmic sound with one’s own body: hand claps of different shapes, finger snaps, pats on the chest and thighs, stamps and, in some traditions, vocal percussion. It is found in many musical traditions (Section 2.5) and is increasingly used as an intervention. In schools it serves to teach rhythm and musical literacy, to support language learning and to train attention and executive control. Hand-clapping to the rhythm of newly learned words has been reported to improve second-language pronunciation (Zhang et al., 2020), and spontaneous hand-clapping songs have been linked to aspects of child development (Brodsky and Sulkin, 2011). In health and community settings, body percussion appears in programmes for healthy ageing and for children with attention-deficit/hyperactivity disorder (Romero-Naranjo and Liendo-Cárdenas, 2025; Romero-Naranjo and Navarro-Camacho, 2026a), and it shares with rhythmic auditory stimulation the premise that rhythm can organise movement (Thaut et al., 1996). It also sits at the border between music and dance: in tap, *zapateado*, gumboot and step dancing the moving body is at the same time the instrument. This article argues that the label “body percussion” covers interventions that differ in their plausible active ingredients, and that these ingredients, rather than the label, must be reported if the evidence is to be synthesised and applied.

The practices that share the label are diverse. A bibliometric survey identified 245 documents on body percussion indexed between 2001 and 2022 and found education to be its main field of application (Arnau-Mollá and Romero-Naranjo, 2024). Body percussion pedagogy is not a single method. Several approaches have developed in parallel, among them body music, a term introduced by Keith Terry, and the KeKeÇa approach in Türkiye, whose founder trained as an Orff-Schulwerk teacher trainer and which has been used to prepare primary-school teachers to teach curriculum content through body music games (Bulut et al., 2025). Even within a single method, programmes described as body percussion range from seated clapping sequences to stepping routines with ankle weights, elastic bands and balls aimed at physical fitness (Romero-Naranjo and Andreu-Cabrera, 2023; Andreu-Cabrera et al., 2024). The label can also conceal the very contrast a study is designed to test. A recent four-arm study in Costa Rican primary education compared a dual-task body percussion programme with standard physical education, with a second body percussion arm limited to repetitive, pre-set choreographies, and with a passive control (Romero-Naranjo and Navarro-Camacho, 2026b). Both body percussion arms involved clapping and patting to music, but they were designed to differ in cognitive load and in the extent to which movements could become automatic. A systematic review that coded both as “body percussion” would pool an intervention with one of its own comparators. Dance research has already met this problem: meta-analyses of dance interventions for older adults have pooled programmes of different styles, frequencies and durations (Meng et al., 2020; Hewston et al., 2021), so the features that drive any effect cannot be identified from the pooled estimate. When studies of a practice are reported mainly under the names of programmes, the name tends to stand in for a description of what participants actually did. A reader who knows the programme can reconstruct the intervention; a systematic reviewer, clinician or teacher who does not know it cannot.

These differences matter because they are not neutral variations in method. Research on bimanual coordination, sensorimotor synchronisation, dual-task performance and interpersonal synchrony gives grounds for expecting each of them to change what participants do and, plausibly, what they gain; Section 2 sets out this evidence feature by feature, and Table 1 summarises it. Direct evidence from body percussion research is still scarce. The clearest case concerns second-language phonology: in a coding of eleven studies of rhythmic-motor training, the four studies in which movements were fully aligned with linguistic events reported gains on the target feature and the three unaligned studies did not, a pattern that rests on few studies, was coded by a single rater and is confounded with research group (Romero-Naranjo, 2026e). For other features, such as the provenance of repertoire and physical contact, the case for reporting rests mainly on replicability, safety and ethics. If the differences between programmes were merely methodological variation, a synthesis could average over them; because they are candidate active ingredients, a synthesis needs to model them, and it can do so only if they are reported.

**Table 1:** Why the features prompted by BODYPERC are expected to matter: hypothesised route to outcomes and type of supporting evidence.

Feature (BODYPERC items)	Hypothesised route to outcomes, replicability or safety	Key sources	Type of support
Sound sources, mediation and posture (items 4, 5, 7)	Determine the motor task, balance demand, impact load and risk profile; preferred tempo may depend on the effector	Alonso-Marco and Romero-Naranjo, 2022; Romero-Naranjo, 2026b	D (preliminary, single performer); H; R (safety)
Interlimb organisation (item 6)	In-phase, symmetric patterns are the most stable; alternating (antiphase) patterns are less stable and destabilise as frequency rises; practice and attention to the integrated pattern help to overcome these constraints	Kelso, 1984; Swinnen and Wenderoth, 2004	N
Demonstration and transmission mode (items 6, 12)	Children imitating a facing model reproduce the goal rather than the exact movement; anatomical imitation is proposed to require an additional spatial transformation; the route by which a pattern is learned may change its cognitive demand	Bekkering et al., 2000; Romero-Naranjo, 2026d	N; H
Tempo (item 8)	Timing variability depends on tempo; tempo determines whether a given interlimb pattern remains stable	Repp and Su, 2013; Kelso, 1984	N
Metric and pattern structure (item 9)	Structural features of clapping patterns predict how difficult they are to perform	Duffy and Pearce, 2018; Repp and Su, 2013	D (performance, not intervention outcomes); N
Alignment of strokes with linguistic units (item 10)	In a coding of eleven studies, rhythmic-motor training aligned with linguistic events was associated with phonological gains and unaligned training was not	Romero-Naranjo, 2026e	D (preliminary: eleven coded studies, single coder)
Repertoire provenance (item 13)	Identifies the material for replication; makes cultural borrowing visible; gendered transmission may affect participation	Marsh, 2008; Doubleday, 2008	R
Concurrent task, priority and progression (items 14–16)	Dual-task interference depends on the type of concurrent task and on the priority instruction; executive gains appear to require progressively increased demand	Al-Yahya et al., 2011; Plummer and Eskes, 2015; Diamond and Ling, 2016	N
Unit of delivery, coordination demand and formation (items 17, 18)	Synchrony increases prosocial behavior and bonding, directed toward synchronous partners; the distribution of influence within the group may shape exploration and transfer	Rennung and Göritz, 2016; Mogan et al., 2017; Cirelli et al., 2014; Romero-Naranjo, 2026h	N; H
Physical contact (item 19)	Consent, assent and tolerability; contact may itself modify social outcomes	Piper and Smith, 2003; Romero-Naranjo, 2026a	R; H
Session structure and schedule (items 20, 21)	Exposure is active playing time, not timetabled time; distributed practice and practice scheduling affect learning	Cepeda et al., 2006; Shea and Morgan, 1979; Pope, 2016	N

D, direct evidence from body percussion or clapping research; N, evidence from neighbouring fields (bimanual coordination, sensorimotor synchronisation, dual-task, interpersonal synchrony, motor learning) that has not yet been tested as a moderator of body percussion intervention outcomes; H, hypothesis proposed but not yet tested; R, reason of replicability, safety or ethics that does not depend on a mechanism. Apart from the preliminary coding of alignment in second-language studies, no feature has yet been tested as a moderator of outcomes across body percussion interventions; consistent reporting is the precondition for such tests.

This is a specific instance of a well-documented problem. Descriptions of non-pharmacological interventions in trials and systematic reviews often omit the information needed to replicate them (Glasziou et al., 2008), and in a consecutive sample of randomised trials fewer than half of the non-pharmacological interventions were adequately described in the primary publication (Hoffmann et al., 2013). Reporting of music interventions in healthcare is similarly inconsistent (Robb et al., 2018). Poor description limits synthesis and implementation: reviews cannot group like with like, meta-analyses pool interventions that share a label but not an active ingredient, and practitioners cannot implement what they cannot see (Hoffmann et al., 2017).

Study design matters as well. Allocation concealment, blinding and the use of researcher-developed measures shape the effect estimates that studies report (Wood et al., 2008; Cheung and Slavin, 2016), and conclusions about cognitive training often depend on what the intervention is compared with (Diamond and Ling, 2016; Sala and Gobet, 2020). The reporting gap is also visible in the authors’ own research group: a quasi-experimental study of selective attention acknowledged as a limitation that the programme followed by each group could have been presented in more detail (Romero-Naranjo et al., 2023). What distinguishes one body percussion intervention from another, and therefore what a comparison can show, becomes visible only when reports describe the intervention, the comparator and the delivery conditions feature by feature.

Existing reporting guidelines address part of this need. The Template for Intervention Description and Replication (TIDieR) provides twelve items applicable to any intervention (Hoffmann et al., 2014); the Consensus on Exercise Reporting Template (CERT) adds sixteen items on exercise content, dose and progression (Slade et al., 2016); the reporting guidelines for music-based interventions (Robb et al., 2011) were recently updated through a Delphi process, and the resulting 12-item RG-MBI checklist is presented with an explanation and elaboration guide (Robb et al., 2025); CONSORT, SPIRIT and CONSORT-SPI govern the reporting of randomised trials and their protocols (Montgomery et al., 2018; Chan et al., 2025; Hopewell et al., 2025); and TREND covers non-randomised evaluations (Des Jarlais et al., 2004). None of them, however, prompts the features that make one body percussion intervention different from another: which body surfaces are struck, how the limbs are organised relative to one another, at what tempo and with what rhythmic structure, under what concurrent cognitive load, in what social formation and with what repertoire. These are the candidate active ingredients described above.

The aim of this article is to propose BODYPERC, a checklist for reporting body percussion interventions and their comparators. The proposal has three constraints. First, it is method-neutral: no item presupposes a particular pedagogy, and every item can be completed for a school hand-clapping game, a flamenco *palmas* workshop, a clinical rhythm programme or a proprietary method. Second, it extends rather than replaces existing guidance and is designed to be used with TIDieR and, where relevant, CERT, CONSORT or TREND. Third, it is provisional: it derives from conceptual analysis rather than expert consensus, and the steps needed to turn it into a consensus-based standard are set out in the Discussion. BODYPERC is not a tool for appraising study quality and does not recommend any intervention or method.

## 2 Body percussion as an intervention class: what generic guidelines do not prompt

An intervention class needs its own reporting extension when its active ingredients are not captured by a generic template. Five features distinguish body percussion from exercise programmes, music listening and instrumental music training, and together they justify an extension. The subsections that follow explain why each feature is expected to influence outcomes, replicability or safety. Table 2 shows how far TIDieR, CERT and the RG-MBI checklist already cover these features, and Table 1 summarises the hypothesised route from each feature to outcomes and the type of evidence that supports it.

**Table 2:** Coverage of body percussion features by existing intervention-reporting guidelines.

Feature of a body percussion intervention	TIDieR	CERT	RG-MBI	BODYPERC item(s)
Feature-based description independent of a method’s name	Partial	Partial	Partial	1
Link between hypothesised mechanism and outcome	Partial	No	Yes	2
Function of the practice and domain of application	No	No	No	3
Sound sources, body surfaces, degree of mediation	Partial	Partial	Partial	4, 5
Interlimb organisation, laterality, demonstration mode	Partial	Partial	No	6
Posture, support and locomotion	Partial	Partial	No	7
Tempo, metre and pattern structure	No	No	Partial	8, 9
Simultaneous voice, singing or speech	No	No	Yes	10
Auditory context and cueing	No	No	Yes	11
Transmission mode (imitation, syllables, notation)	Partial	No	Partial	12
Provenance of repertoire	No	No	Partial	13
Concurrent cognitive task, load level, priority	No	No	No	14, 15
Progression rules	Partial	Yes	No	16
Group size and ratio	Partial	Partial	Yes	17
Coordination demand and spatial formation	No	No	No	18
Physical contact, consent and refusal	No	No	No	19
Session structure and active playing time	Partial	Partial	Partial	20
Frequency, duration, time of day and spacing	Partial	Partial	Partial	21
Provider qualifications and training	Yes	Yes	Yes	24
Function held constant and form allowed to vary	Partial	Partial	Partial	26
Comparator type and equivalence audit	Partial	No	No	28, 29
Fidelity planned and achieved	Yes	Yes	Yes	30, 31
Process measures from the sound (timing, synchrony, dual-task cost)	No	No	No	32
Adverse events and tolerability	No	Yes	No	33

Yes, the guideline contains an item that prompts the feature; Partial, a generic item could accommodate the information but does not prompt it; No, not addressed. Cell shading mirrors these ratings and is provided only to aid reading; the BODYPERC column is not shaded. TIDieR, Template for Intervention Description and Replication (Hoffmann et al., 2014); CERT, Consensus on Exercise Reporting Template (Slade et al., 2016); RG-MBI, Reporting Guidelines for Music-based Interventions, rated in their updated version (Robb et al., 2025). Items 22, 23, 25 and 27, adopted from existing guidelines without elaboration, are not listed. Ratings are the authors’ judgement and are offered for scrutiny in the consensus stage.

### 2.1 The effector is the instrument

In instrumental music the performer’s movement and the sounding object are distinct: the drum can be described without describing the drummer. In body percussion they coincide. The sound is the acoustic trace of a specific movement of a specific body segment, so describing *what is played* also prescribes *what the body does*. The Hornbostel–Sachs system classifies instruments according to what vibrates to produce the sound (von Hornbostel and Sachs, 1961) and does not readily accommodate the human body as a sound source. A recent biaxial taxonomy addresses this gap by separating two questions that linear classifications conflate (Romero-Naranjo and Di Russo, 2026). An organological axis describes the sound source and the degree of mediation between gesture and sound: endogenous (body on body), environmental (body on floor, table or object), clothing-based or prosthetic (shoes, gumboots, cups, coconut shells) and electroacoustic (sensors that trigger sound). A functional axis describes what the practice is for, for example compositional, accompanying, pedagogical, occupational, ritual, performative or therapeutic purposes.

Sound sources and the degree of mediation must therefore be named, because they determine the motor task. A pattern played on the thighs while seated and the same pattern played as stamps while standing share a rhythm but not a motor demand, a balance demand or a risk profile. Two further considerations support precise reporting of effectors. A preliminary kinematic analysis of basic body percussion movements, based on a single performer, suggests that they differ in joint excursion and segmental velocity (Alonso-Marco and Romero-Naranjo, 2022); if confirmed in larger samples, such differences could matter for safety in older or clinical populations. In addition, it has been hypothesised that the preferred rate of a rhythmic movement depends partly on the mechanics of the effector that produces it (Romero-Naranjo, 2026b); if so, tempo cannot be interpreted without knowing which limb produced the sound.

Interlimb organisation is a related dimension that generic templates do not prompt. Body percussion patterns vary in whether the hands move symmetrically, alternately or independently, whether they cross the body midline, and how many independent motor streams (hands, feet, voice) run at once. These distinctions correspond to well-documented constraints on coordination. Symmetric, in-phase movement of the two hands is the most stable mode; alternating, antiphase patterns are less stable and, as movement frequency increases, tend to switch abruptly to the in-phase mode (Kelso, 1984). Such constraints can be overcome with practice, particularly when attention is directed to the integrated pattern rather than to each hand separately (Swinnen and Wenderoth, 2004), which is what many body percussion progressions require. Interlimb organisation and tempo therefore jointly determine the motor demand of a pattern, and a report that states one without the other underspecifies it. The way a movement is demonstrated also matters. A teacher facing the group produces mirror imitation, in which the teacher’s left corresponds to the learner’s right. Imitating a model who faces the learner is not a literal copy: pre-school children imitating gestures made by an experimenter sitting opposite them reproduced the goal of the gesture, such as the ear to be touched, but often changed the hand used to reach it (Bekkering et al., 2000). Anatomical imitation requires an additional spatial transformation and is cognitively more costly than mirror imitation (Romero-Naranjo, 2026d). Demonstration mode is therefore a determinant of the task rather than a stylistic detail.

### 2.2 Rhythmic structure is a dose parameter

In exercise interventions, dose is expressed as frequency, intensity, time and type (Garber et al., 2011). In body percussion, part of the dose is carried by the rhythmic content. Tempo, pattern length, metre, subdivision, syncopation and the placement of rests all change the difficulty of a pattern. Performance data from a large sample of players of a game based on Steve Reich’s *Clapping Music* show that structural features of a clapping pattern predict how hard it is to perform (Duffy and Pearce, 2018), and the sensorimotor synchronisation literature documents that timing variability depends on tempo and rhythmic complexity (Repp and Su, 2013). Two programmes with identical session length and frequency can therefore impose very different demands if one works at 60 beats per minute with four-beat unison patterns and the other at 120 beats per minute with syncopated interlocking parts. The updated music-intervention guidelines ask for compositional features such as tempo and rhythm to be reported where relevant (Robb et al., 2025), but they treat these as properties of the music, not as a motor prescription that participants execute with their own bodies.

### 2.3 Cognitive load is deliberately manipulated

Many body percussion programmes add a concurrent task to a pattern that participants already know. Participants clap while counting backwards, stamp while answering a question, or switch pattern at an unpredictable signal. Such activities are dual tasks, and the size of dual-task interference depends on the type of concurrent task (Al-Yahya et al., 2011). A coherent taxonomy of concurrent tasks is needed for results to be comparable across studies (McIsaac et al., 2015), and research on multitasking shows that interference depends on the structure of the tasks and on practice (Koch et al., 2018). Rhythm has a particular status in this literature. A rhythmic tapping task has been proposed as a secondary task for measuring cognitive load (Park and Brünken, 2015), rhythm–motor tasks have been examined as a dual-task paradigm for older adults (Kim et al., 2017), and dual-task drum playing with rhythmic cueing has been piloted in people with Parkinson’s disease (Park and Kim, 2021). Interference must also be measured in both directions: a participant can preserve the motor pattern by abandoning the secondary task, or the reverse, and a report of motor performance alone can hide this trade-off (Plummer and Eskes, 2015).

Because gains in executive function appear to depend on demands that are progressively increased rather than held constant (Diamond and Ling, 2016), the level of load and the rule for raising it are part of the dose rather than incidental detail. Reports therefore need to specify the concurrent task, the instruction about which task to prioritise and the level of load at which each activity was delivered. An ordinal scale can make load comparable across activities. One recent proposal defines six levels by the manipulation superimposed on a stable base pattern (guided execution, removal of the model, memory load, inhibition load, switching load and a heterogeneous concurrent task from another domain) and treats their ordering as a hypothesis to be tested with Rasch models rather than as a definition (Romero-Naranjo, 2026f). BODYPERC does not require that particular scale. It requires that some explicit scale be used and reported, and that the executive demand attributed to each level be interpreted with the caution recommended by the unity-and-diversity model of executive functions (Miyake et al., 2000).

### 2.4 Interpersonal synchrony is a social ingredient

Body percussion is usually practised in groups, and moving in time with others is itself a plausible active ingredient. Meta-analyses indicate that interpersonal synchrony increases prosocial behavior and perceived social bonding (Rennung and Göritz, 2016; Mogan et al., 2017), and joint rhythmic movement has been shown to increase sharing and fairness among four-year-olds (Rabinowitch and Meltzoff, 2017). Already in infancy, the increase in helping that follows synchronous movement is directed toward the partner with whom one moved (Cirelli et al., 2014). If synchrony contributes to outcomes, the social format of an intervention is part of its content. The relevant features include the spatial formation (circle, rows, facing pairs), the coordination demand between participants (unison, canon, call and response, interlocking parts, leader–follower exchanges or improvisation) and group size. The source of timing may also matter. It has been proposed that whether participants follow a leader or a recording they cannot influence, or adjust mutually to one another, shapes the range of coordination solutions a group explores and its transfer to untrained patterns (Romero-Naranjo, 2026h). The proposal has not yet been tested, but it implies that unison led from the front and interlocking parts without a leader are different interventions. TIDieR asks whether an intervention was delivered individually or in groups (Hoffmann et al., 2014), and CERT asks the same of exercise (Slade et al., 2016), but neither asks how participants had to coordinate with one another.

Group body percussion also frequently involves touch: hands meet in paired clapping games, and participants hold or brush against one another in circle formations. Touch raises questions of consent, particularly in asymmetric relationships such as teacher and pupil (Piper and Smith, 2003), and of tolerability for people with tactile hypersensitivity. A graduated approach to contact with explicit opt-out procedures has been proposed for educational settings (Romero-Naranjo, 2026a). Whether contact occurs, and how consent and refusal are handled, should be reported for ethical reasons and because contact may itself modify social outcomes.

### 2.5 Repertoire comes from living traditions and is delivered by non-clinicians

Body percussion repertoire is often drawn from, or modelled on, living traditions: children’s hand-clapping games transmitted across playgrounds and continents (Marsh, 2008), flamenco palmas, South African gumboot dancing, Bavarian Schuhplattler or African American hambone. These are what Mauss called techniques of the body, socially transmitted ways of using the body (Mauss, 1973). Their use in interventions raises questions that generic guidelines do not anticipate: where the material comes from, how it was adapted, whether its source is acknowledged and whether its social meanings travel with it. Gender is one such meaning. Hand-clapping games are transmitted predominantly among girls (Marsh, 2008), and the association between musical instruments, gender and access is well documented (Doubleday, 2008). The sex composition of groups and the provenance of repertoire can therefore bear on both participation and outcomes. For repertoire, unlike the features discussed above, the case for reporting rests mainly on replicability and ethics rather than on a demonstrated mechanism: no study known to the authors has compared outcomes across repertoires matched on the other features.

Body percussion interventions are often delivered by music teachers, physical education teachers, dance instructors and therapists rather than by clinicians following a manual, which makes fidelity both more important and harder to secure. Outcomes depend on how well a programme is delivered (Durlak and DuPre, 2008), and fidelity must be balanced against adaptation to local cultures and settings (Castro et al., 2004). One influential resolution is to standardise the *function* of each component while allowing its *form* to vary (Hawe et al., 2004). This applies directly to body percussion, where the same function, such as sustaining a pulse under a concurrent verbal task, can be realised with repertoire from different traditions. A reporting framework must therefore make it possible to state which features were fixed and which were free to vary.

## 3 Development of the checklist

Moher et al. (2010) describe a sequence for developing reporting guidelines that begins with identifying the need for a guideline, reviewing the literature and generating a candidate item list, and continues through a consensus process, pilot testing, publication of an explanation-and-elaboration document and evaluation of uptake. The present article completes the first stages of that sequence and stops before consensus. BODYPERC should accordingly be read as a structured candidate item list offered for scrutiny.

Items were generated in four steps. First, the intervention features addressed by TIDieR (Hoffmann et al., 2014), CERT (Slade et al., 2016), the music-intervention reporting guidelines (Robb et al., 2011, 2025), the TIDieR extensions for population health and for placebo and sham controls (Campbell et al., 2018; Howick et al., 2020), and frameworks of treatment and implementation fidelity (Dane and Schneider, 1998; Bellg et al., 2004; Carroll et al., 2007) were inventoried. Second, the features of body percussion practice described in Section 2 were analysed, drawing on organology, the sensorimotor-synchronisation and dual-task literatures, research on interpersonal synchrony and ethnomusicology. Third, each body percussion feature was mapped onto the existing items and classified as *adopted* (an existing item covers it adequately), *extended* (an existing item covers it but needs body-percussion-specific elaboration) or *new* (no existing item prompts it). Fourth, each candidate item was retained only if it satisfied four drafting rules.

The drafting rules were as follows. (i) *Observability*: the item must be answerable from information available to the investigators, without access to proprietary training materials. (ii) *Method neutrality*: the item must describe a feature of the intervention without endorsing or presupposing a pedagogy, and it must be completable for traditional, educational, clinical and proprietary forms of body percussion alike. (iii) *Relevance*: there must be a plausible argument that the feature affects outcomes, replicability, safety or interpretation. (iv) *Non-redundancy*: the item must not duplicate information already required by CONSORT, SPIRIT, CONSORT-SPI or TREND, such as randomisation procedures, blinding or sample-size calculation. Items were then classified as *core*, applicable to every body percussion intervention, or *contextual*, applicable when the feature is present (for example, physical contact or home practice).

Method neutrality was treated as a design requirement for two reasons. The first is scientific: a guideline that describes interventions in the vocabulary of one method makes it harder to compare that method with others. The second concerns transparency. The first author developed the BAPNE body percussion method and both authors teach it, and a reporting framework proposed by those who develop and teach an intervention could be suspected of favouring it. Three safeguards were adopted. Every item is phrased in terms of observable features, and a method’s name is reported only under item 1, where TIDieR already asks for a brief name, and never replaces the description. The examples in this article are drawn from several traditions and settings. The consensus stage described in Section 7 is designed to be led by an independent, multidisciplinary panel. The interest is declared in the Conflict of Interest statement.

## 4 The BODYPERC checklist

BODYPERC comprises 33 items organised in 11 domains (Table 3; Figure 1). Thirteen items are new, eleven extend an existing item and nine are adopted from existing guidelines; 26 are core and seven contextual. The domains follow a simple logic. After identification and rationale (domain 1), four domains describe what is played and how: sound production (domain 2), rhythmic and musical structure (domain 3), cognitive load (domain 4) and social format (domain 5). Three domains describe how much, by whom and with what flexibility: dose and schedule (domain 6), providers and setting (domain 7), and tailoring and modification (domain 8). The last three describe against what and how well: the comparator (domain 9), fidelity and process measures (domain 10), and safety (domain 11). The following subsections explain the rationale for the new and extended items; adopted items are explained in the guidelines from which they come.

**Table 3:** The BODYPERC checklist for reporting body percussion interventions and their comparators (version 0.1, for consultation).

No.	Item	What to report	Origin	Status
Domain 1. Identification and rationale				
1	Name	Brief name. If a named method or programme is followed: name, version, source. The name never replaces the feature description (items 4–33).	E · TIDieR 1	Core
2	Rationale and mechanism	Each hypothesised mechanism (e.g., sensorimotor timing, inhibitory control, coordination, physical activity, social bonding, affect regulation, phonological processing) and the outcome chosen to test it.	E · TIDieR 2	Core
3	Domain and function	Application domain (educational, clinical, rehabilitative, community, performance). For imported repertoire, its function in the source tradition (e.g., accompanying, occupational, ritual, pedagogical).	N	Core
Domain 2. Sound production				
4	Sound sources	All body surfaces struck and stroke types (e.g., flat/cupped claps, chest or thigh pats, stamps, snaps, vocal percussion); approximate share of playing time for each.	N	Core
5	Degree of mediation	Endogenous, environmental, clothing-based/prosthetic or electroacoustic; objects, footwear, props or sensors used.	E · TIDieR 3; CERT 1	Core
6	Interlimb organisation	Symmetric, alternating or independent limb movement; midline crossing; maximum number of concurrent independent motor streams (hands, feet, voice); mirror or anatomical demonstration.	N	Core
7	Posture and locomotion	Seated, standing, stepping or travelling; support available; changes across the programme.	E · CERT 8	Core
Domain 3. Rhythmic and musical structure				
8	Tempo	Beats per minute (or inter-onset interval); fixed, progressive or self-selected; how set (metronome, recording, leader, participant); range across the programme.	N	Core
9	Metric and pattern structure	Metre, cycle length, subdivision, syncopation, polyrhythm, placement of rests; notated or video examples of representative patterns.	N	Core
10	Voice and language	Simultaneous singing, speech, counting or rhythmic syllables; language; whether text carries curricular content; temporal alignment of strokes with linguistic units.	E · RG-MBI 3b	*Contextual*
11	Auditory context	Live music, recorded music, metronome or silence; playback system where relevant.	A · RG-MBI 3c	Core
12	Transmission mode	Imitation (mirror/anatomical), oral mnemonics or syllables, notation (which system), video.	N	Core
13	Repertoire provenance	Traditional, adapted, newly composed or taken from a method; source communities acknowledged.	N	*Contextual*
Domain 4. Cognitive load				
14	Concurrent tasks and load	Type and exact content of each concurrent task (verbal, numerical, visuospatial, memory, inhibition, switching); level on an explicit ordinal load scale; or a statement that none was used.	N	Core
15	Priority instruction	What participants were told about priority between the rhythm and the concurrent task.	N	*Contextual*
16	Progression	Variable increased (tempo, pattern length, motor streams, load level), criterion for progressing, group or individual progression.	E · CERT 7a–7b	Core
Domain 5. Social format				
17	Unit of delivery	Individual, dyad or group; group size; participant-to-provider ratio.	A · RG-MBI 4, 5; TIDieR 6	Core
18	Coordination demand	Unison, canon, call and response, interlocking parts, leader–follower, improvisation; spatial formation (circle, rows, facing pairs); source of timing (leader, recording or mutual adjustment).	N	Core
19	Physical contact	Whether contact occurs, between whom, how it is graduated; consent, assent and refusal procedures; accommodations for tactile hypersensitivity.	N	*Contextual*
Domain 6. Dose and schedule				
20	Session structure	Phases and planned minutes; active playing time distinguished from instruction, demonstration and transitions.	E · CERT 13	Core
21	Schedule	Frequency, session length, weeks, total sessions; time of day; spacing between sessions.	E · TIDieR 8	Core
22	Physical intensity	Heart rate, perceived exertion or accelerometry; rest intervals.	A · CERT 13	*Contextual*
23	Independent practice	Whether prescribed, content, and how monitored.	A · CERT 9	*Contextual*
Domain 7. Providers and setting				
24	Providers	Profession; musical and movement expertise; hours and format of training in the intervention; certification; whether the provider also administered outcome assessments.	E · TIDieR 5; CERT 2	Core
25	Setting and materials	Location and space; delivery mode (face to face, video, online); where materials, scores and videos can be accessed.	A · TIDieR 3, 6, 7	Core
Domain 8. Tailoring and modification				
26	Planned tailoring	Components whose function was held constant and components whose form could vary (e.g., repertoire, language); adaptations for age, disability or sensory needs.	E · TIDieR 9	Core
27	Unplanned modifications	What changed during the study, when and why.	A · TIDieR 10	Core
Domain 9. Comparator				
28	Comparator type and content	Type from an explicit taxonomy (passive, wait-list, usual practice, attention-matched, active non-rhythmic movement, active rhythmic without body sound, dismantling, graded active, within-participant, historical); content described with BODYPERC items where applicable; or a statement that no comparator was used.	E · CONSORT-SPI; TIDieR-Placebo	Core
29	Equivalence audit	Evidence of equivalence on dose received, temporal distribution, provider, setting and materials, group size, participant expectation (verbatim presentation text) and perceived effort and enjoyment.	N	Core
Domain 10. Fidelity and process				
30	Fidelity plan	Framework and dimensions (e.g., adherence, exposure, quality, responsiveness, differentiation); monitoring methods; raters; reliability.	A · TIDieR 11; CERT 16a	Core
31	Fidelity achieved	Dose delivered and received (attendance, active minutes); proportion of sessions meeting criteria; differentiation from the comparator.	A · TIDieR 12; CERT 16b	Core
32	Process measures	Accuracy, timing precision (ms), stability, interpersonal synchrony and bidirectional dual-task cost, from audio, video or sensors.	N	*Contextual*
Domain 11. Safety				
33	Adverse events	Pain, skin irritation, fatigue, falls, distress from sound or touch; how monitored; explicit statement if none occurred.	A · CERT 11	Core

Origin: N, new item; E, extends the cited item; A, adopted from the cited guideline. Core items apply to every body percussion intervention; contextual items apply when the feature is present, and their absence should be stated. Where a comparator is used, items 3–27 should be completed for it as well (item 28). BODYPERC is intended for use with TIDieR and, where relevant, CERT, CONSORT, CONSORT-SPI, TREND and SPIRIT; it does not repeat their trial-design items.

**Figure 1:**
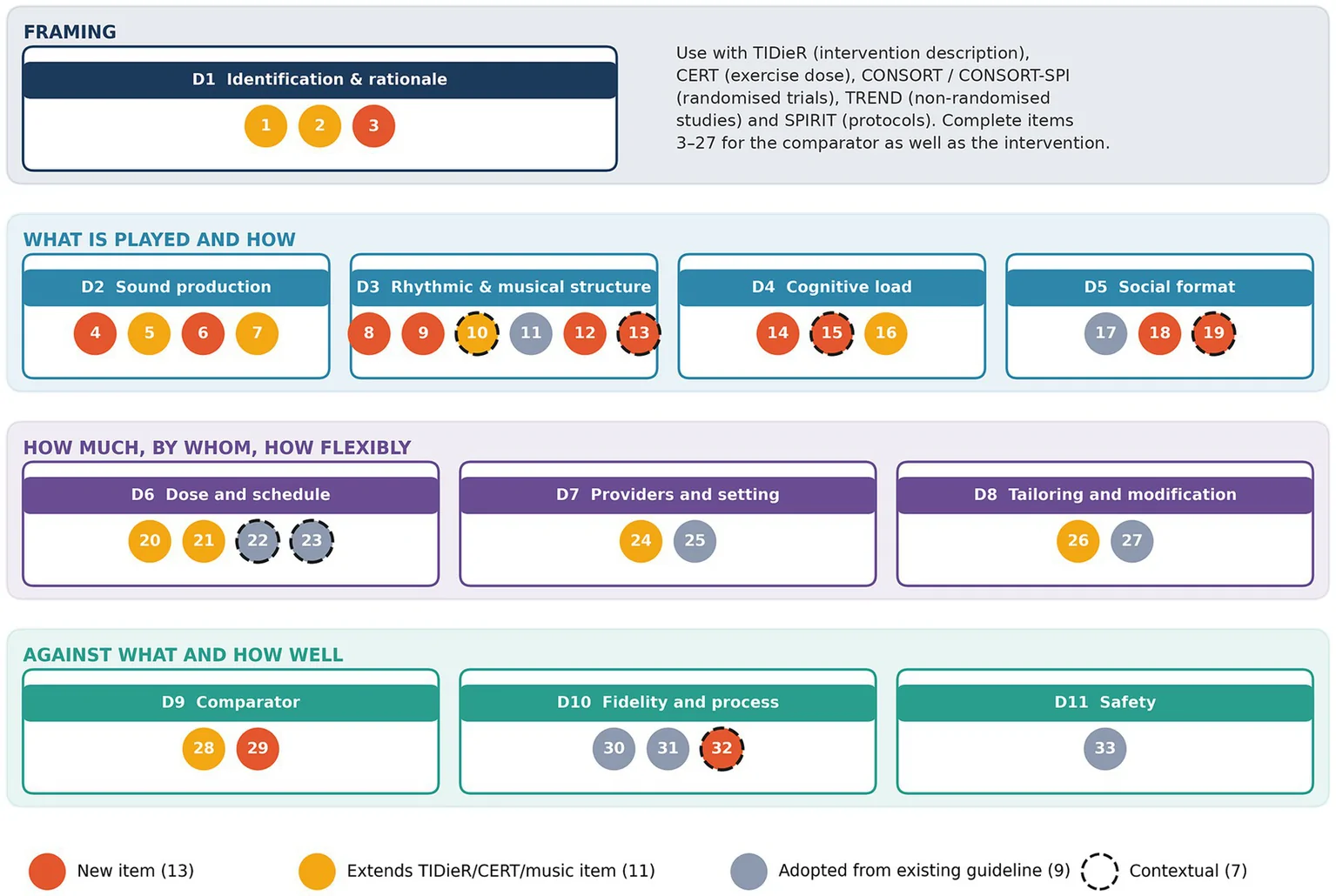
Architecture of the BODYPERC checklist. The 33 items are grouped into 11 domains arranged in four blocks: framing, what is played and how, how much, by whom and how flexibly, and against what and how well. Each circle is one item, numbered as in Table 3. Colour indicates origin: new items (no existing guideline prompts the feature), extended items (an existing TIDieR, CERT or music-intervention item is elaborated for body percussion) and adopted items (taken from an existing guideline). Items marked with a ring are contextual, meaning they are reported only when the feature is present.

### 4.1 Identification and rationale (items 1–3)

Item 1 extends TIDieR’s request for a brief name. When an intervention follows a named method or programme, the name, version and source should be given, but the name must never replace the feature description required by the remaining items. The principle is the one behind taxonomies of behavior-change techniques, which describe interventions by their active components rather than by their labels (Michie et al., 2013). Item 2 extends TIDieR’s rationale item by requiring an explicit link between each hypothesised mechanism and the outcome chosen to test it. Body percussion is credited with effects through several routes: sensorimotor timing, executive control, motor coordination, physical activity, social bonding, emotional regulation and phonological processing. A report that measures inhibitory control should say why the intervention is expected to act on inhibition rather than on attention in general. This mapping, recommended for complex interventions generally (Skivington et al., 2021), is also what allows a null result to be informative. Item 3 asks for the application domain (educational, clinical, rehabilitative, community, performance training) and, where repertoire is imported from a tradition, the function it had in that tradition, using a functional classification such as the biaxial taxonomy described above (Romero-Naranjo and Di Russo, 2026).

### 4.2 Sound production (items 4–7)

Item 4 asks for every sound source used, with the body surfaces struck and the type of stroke (for example, flat or cupped claps, chest pats, thigh pats, heel or flat-foot stamps, snaps, vocal percussion), and, where feasible, the approximate proportion of playing time spent on each. Item 5 extends TIDieR’s materials item and CERT’s equipment item to the degree of mediation: whether the sound is endogenous, environmental, clothing-based or prosthetic, or electroacoustic, and which objects or garments were used. Item 6 is new and concerns interlimb organisation: symmetric, alternating or independent limb movements, midline crossing, the maximum number of independent motor streams running concurrently, and whether the provider demonstrated in mirror or anatomical orientation. Item 7 extends CERT’s exercise description to posture, support and locomotion, that is, seated, standing, stepping or travelling through space, and whether support was available. This item is essential for interpreting falls risk and physical-activity outcomes in older and clinical populations.

### 4.3 Rhythmic and musical structure (items 8–13)

Items 8 and 9 are the core of what makes body percussion reporting distinct. Item 8 requires tempo in beats per minute (or inter-onset interval), stating whether it was fixed, progressive or self-selected, how it was set (metronome, recording, live leader, participant), and its range across the programme. Item 9 requires the metric and pattern structure: metre, cycle length, subdivision, syncopation, polyrhythm and the placement of rests. Notated examples, or a link to notated or video examples, should be provided for representative patterns, because a verbal description of rhythm is almost always ambiguous. Item 10 extends the music-intervention guidance to simultaneous voice use, covering singing, speech, counting or syllables, the language used, whether the text carried curricular content and, when language outcomes are targeted, whether strokes were temporally aligned with linguistic units such as stressed syllables, a feature proposed to moderate language outcomes (Romero-Naranjo, 2026e). Item 11 is adopted from the same guidance and concerns the auditory context: live music, recorded music, metronome or silence, and the playback system where relevant.

Item 12 is new and asks how patterns were transmitted: by imitation (mirror or anatomical), through oral mnemonics or rhythmic syllables, through notation (and which notation system), or through video. Some traditions name each sound source with a syllable; in the KeKeÇa vocabulary, for example, separate syllables denote finger snaps, claps, thigh pats, chest pats and steps (Bulut et al., 2025). Where such a vocabulary is used, reporting it together with its mapping to body surfaces makes patterns replicable in writing. Transmission mode may change the cognitive route by which a pattern is learned and is therefore a plausible moderator of both motor and cognitive outcomes. Item 13 is new and concerns the provenance of the repertoire: whether it was traditional, adapted from a tradition, composed for the intervention or taken from an existing method, with an acknowledgement of source communities where applicable. This item serves replication, because it identifies the material, and it also serves an ethical purpose, because it makes cultural borrowing visible. It is contextual because some interventions use newly composed material only.

### 4.4 Cognitive load (items 14–16)

Item 14 asks for every concurrent task, with its type (verbal, numerical, visuospatial, memory, inhibition, switching), its exact content and instruction, and the load level at which each activity was delivered on an explicit ordinal scale. If no concurrent task was used, this should be stated, since the absence of a concurrent task is itself a defining feature. Item 15 is contextual and asks what participants were told about priority between the rhythmic pattern and the concurrent task. Without this information a dual-task cost cannot be interpreted, because the direction of the trade-off depends on the instruction (Plummer and Eskes, 2015). Item 16 extends CERT’s progression items. It asks what variable was increased over the programme (tempo, pattern length, number of concurrent motor streams, load level), what criterion triggered progression, and whether progression was set for the group or for individuals. Progression matters because an activity that becomes automatic may stop exercising what it was meant to train. Recording when each participant reaches each level also yields a process measure that is independent of the outcome tests (Romero-Naranjo, 2026c).

### 4.5 Social format (items 17–19)

Item 17 is adopted from RG-MBI and TIDieR and asks whether delivery was individual, in dyads or in groups, together with group size and the participant-to-provider ratio. Item 18 is new and asks for the coordination demand between participants (unison, canon, call and response, interlocking parts, leader–follower exchanges, improvisation) and for the spatial formation (circle, rows, facing pairs). These features determine how much interpersonal synchrony the intervention demands, which is relevant because synchrony has been associated with prosocial and affiliative outcomes (Mogan et al., 2017). The item also asks whether timing was set by a leader, by a recording or by the participants’ mutual adjustment (Section 2.4). Item 19 is new and contextual. It asks whether patterns involved physical contact between participants or with the provider, how contact was graduated, and what procedures were in place for consent and refusal (Piper and Smith, 2003). Where participants are minors, the report should state how assent was obtained. The item also covers accommodations for tactile hypersensitivity (Romero-Naranjo, 2026a).

### 4.6 Dose and schedule (items 20–23)

Item 20 extends CERT’s dose item to the internal structure of a session: the phases (for example, opening, main activity, closing), the planned minutes for each and the active playing time as distinct from instruction, demonstration and transition time. Two 45-min sessions can contain very different amounts of actual playing, and it is playing time, not timetabled time, that constitutes exposure. Some recent reports already provide a module-by-module table with the planned minutes, content and purpose of each segment of the session (Romero-Naranjo and Navarro-Camacho, 2026b); adding active playing time to such tables would complete the description. Item 21 extends TIDieR’s item on when and how much. It asks for frequency, session length, number of weeks and total sessions, and adds two parameters that affect learning and performance independently of content: time of day and the spacing of sessions. The benefit of distributed over massed practice is well established for verbal learning (Cepeda et al., 2006), the scheduling of practice affects motor learning (Shea and Morgan, 1979), and academic performance has been shown to vary with the time of day at which lessons are scheduled (Pope, 2016). Item 22, adopted from CERT and contextual, asks for physical intensity (heart rate, ratings of perceived exertion such as the Borg scale, or accelerometry; Borg, 1982) when physical or cardiometabolic outcomes are measured or the population is frail. Item 23, also adopted and contextual, asks whether independent practice was prescribed and how it was monitored.

### 4.7 Providers, setting, tailoring and modification (items 24–27)

Item 24 extends TIDieR’s provider item. It asks for the provider’s profession, musical and movement expertise, the amount of training received in the intervention (hours and format) and any certification. It also asks whether the provider administered any outcome assessment. This point is implied by CONSORT’s requirements on blinding, but it deserves its own prompt in school-based research, where the same teacher may both deliver a programme and administer the tests, and where lack of blinding can inflate effect estimates, particularly for subjectively assessed outcomes (Wood et al., 2008). Reporting the assessor is straightforward: one school-based study, for example, specified that every assessment was administered by a neuropsychologist from a general hospital and that the same assessor applied the pre- and post-tests under the same conditions (Romero-Naranjo et al., 2023). Item 25 is adopted from TIDieR and covers setting, delivery mode (face to face, video, online) and where the materials can be accessed. Item 26 extends TIDieR’s tailoring item by asking authors to distinguish, in the sense of Hawe et al. (2004), the components whose function was held constant from those whose form was allowed to vary, for example holding the load level constant while letting teachers choose repertoire from local traditions. Adaptations for disability belong here as well: the bilateral coordination demands of body percussion sequences can be graded, and patterns that assume a standard body may exclude participants with motor impairments, which argues for designing alternative routes from the outset (Romero-Naranjo, 2026g). Item 27, adopted from TIDieR, asks for unplanned modifications during the study.

### 4.8 The comparator (items 28 and 29)

The comparison condition determines what a study is entitled to conclude (Mohr et al., 2009). Item 28 extends the comparator requirements of CONSORT-SPI and TIDieR-Placebo (Montgomery et al., 2018; Howick et al., 2020). It asks for the comparator type, drawn from an explicit taxonomy, and for a description of the comparator using the same BODYPERC items as the intervention wherever they apply. When no comparator is used, this should be stated, since it limits the causal conclusions a study can support. Building on existing classifications of control conditions in behavioral trials (Mohr et al., 2009; Freedland et al., 2011), the taxonomy distinguishes a passive control; a wait-list control; usual practice, whose content varies widely and must be described (Freedland et al., 2011); an attention-matched active control; an active non-rhythmic movement control; an active rhythmic control without body sound, such as instrumental drumming or choreography; a dismantling control, meaning the same intervention minus one component; a set of graded active controls; a within-participant phase design; and a historical comparator (for a fuller treatment in Spanish, see Romero-Naranjo, 2026c). The four-arm design described in the Introduction illustrates the logic: standard physical education, choreographed body percussion and a passive control each address a different rival explanation (Romero-Naranjo and Navarro-Camacho, 2026b). Figure 2 shows which rival explanations each comparator can and cannot rule out.

**Figure 2:**
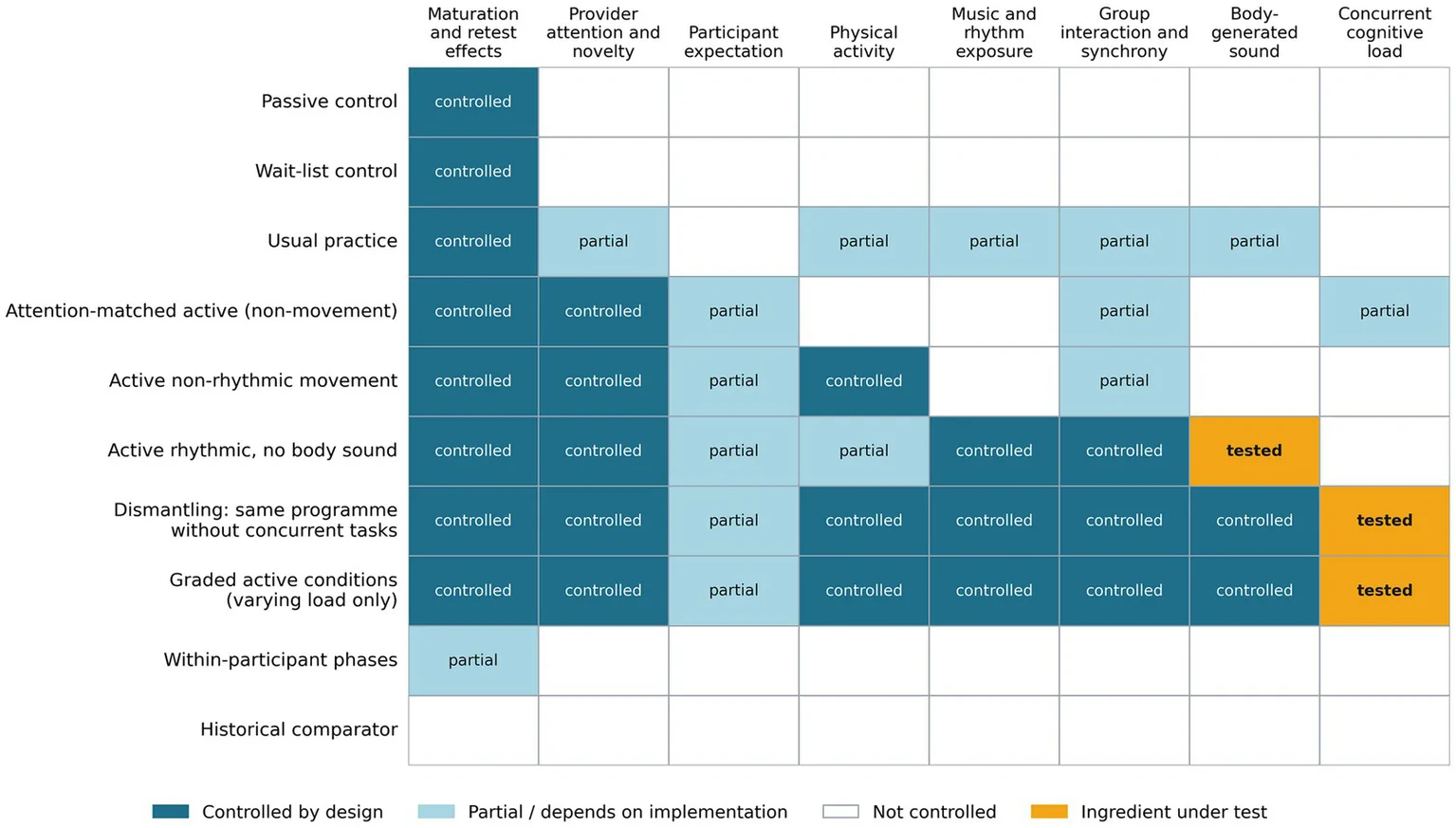
Rival explanations controlled by different comparator types in body percussion research. Rows are comparator types; the first six columns are explanations that compete with a hypothesised active ingredient, and the last two are candidate ingredients in their own right (body-generated sound and concurrent cognitive load). Dark cells: the comparator is designed to control the explanation. Light cells: control is partial or depends on how the comparator is implemented. White cells: not controlled. Amber cells mark the ingredient that the comparator deliberately varies and therefore isolates. The matrix is conceptual; whether a given comparator actually controls an explanation depends on the equivalences documented under item 29. No single comparator controls every explanation, which is why informative designs combine at least one active comparator with a passive one.

Item 29 is new and turns the label “active control” into a claim that can be checked. Calling any alternative activity an active control is a common misuse. An active comparator neutralises a rival explanation only if it is equivalent to the intervention in everything except the ingredient under test, and even then participants’ expectations may differ (Boot et al., 2013). Item 29 therefore asks authors to report, for each comparator, the evidence of equivalence on seven dimensions: total dose actually received, temporal distribution (frequency, time of day, period of the year), provider (experience, training, declared enthusiasm), setting and materials, group size, participant expectation (ideally the verbatim text used to introduce each condition), and perceived effort and enjoyment (Mohr et al., 2009; Boot et al., 2013; Romero-Naranjo, 2026c). Of these, expectation is the most often neglected and potentially the most damaging. In a school, pupils know which group is receiving “the programme,” and no matching of dose can compensate for that. The remedy is not to conceal the study, which would be unethical, but to present every condition as valuable in its own right and to report how this was done.

### 4.9 Fidelity and process measures (items 30–32)

Items 30 and 31 are adopted from TIDieR and CERT and address fidelity as planned and as achieved. Authors should draw on an explicit framework, such as the five dimensions of adherence, exposure, quality of delivery, participant responsiveness and programme differentiation (Dane and Schneider, 1998), the NIH Behavior Change Consortium’s recommendations on design, training, delivery, receipt and enactment (Bellg et al., 2004), or the implementation-fidelity framework of Carroll et al. (2007). They should also report any attendance threshold used to include participants in the analysis, for example attendance at a minimum of 80% of sessions (Romero-Naranjo et al., 2023), because such thresholds change the population to which the results apply. They should report the methods used to monitor fidelity (for example, observation checklists or sampled video), who rated it and with what reliability. For body percussion, differentiation deserves particular attention: when intervention and comparator are both rhythmic or both delivered by the same teachers, contamination is a realistic threat. Process evaluation guidance for complex interventions offers a structure for reporting these data (Moore et al., 2015).

Item 32 is new and contextual, and it reflects an opportunity specific to body percussion. Because the intervention produces sound, its delivery and uptake can be measured from the sound itself. Accuracy (the proportion of strokes with the correct effector and location), timing precision (mean absolute asynchrony relative to the metric grid, in milliseconds), stability (variability of inter-onset intervals) and interpersonal synchrony can be extracted from audio or video recordings using standard sensorimotor-synchronisation methods (Repp and Su, 2013). Under dual-task conditions, a bidirectional dual-task cost, which quantifies change in both the rhythmic and the concurrent task, shows how the imposed load was distributed between the two tasks (Plummer and Eskes, 2015). A proposal for computing such an index in body percussion also addresses the instability of ratio scores at low baseline performance and the lower reliability of difference scores (Romero-Naranjo, 2026f). These measures verify that the intended demand was delivered, characterise the dose received by each participant, and provide candidate mediators linking the intervention to its distal outcomes.

### 4.10 Safety (item 33)

Item 33 is adopted from CERT and asks for adverse events and tolerability, with the procedure used to record them. Body percussion is generally low risk, but it is not risk free. Relevant events include joint or hand pain, skin irritation from repeated striking, fatigue, falls during stepping or travelling patterns, distress in response to loud collective sound or to touch, and exacerbation of pre-existing musculoskeletal conditions. The absence of adverse events should be reported as such, together with how they were monitored, since an unmonitored absence carries no information.

## 5 A worked example

Table 4 illustrates how the new items would be completed for a hypothetical twelve-week programme for community-dwelling older adults, together with its active comparator. The example is constructed rather than drawn from a published study, and its purpose is to show the level of detail each item requires, not to recommend a particular design. It illustrates three points. First, the intervention and the comparator are described with the same items, which makes their differences explicit: here the comparator matches dose, provider, formation and music but removes body sound and concurrent load. Second, the description is complete without reference to any named method. Third, the process measures under item 32 are inexpensive to collect: a single ceiling-mounted microphone per session suffices for the intervention, and wrist-worn accelerometers provide equivalent timing data for the silent comparator. With these measures, a report can document what participants achieved in each session.

**Table 4:** Worked example of the new BODYPERC items for a hypothetical 12-week programme for community-dwelling older adults and its active comparator.

Item	Intervention: body percussion with concurrent tasks	Comparator: rhythmic movement without body sound
3 Domain and function	Community healthy-ageing programme. Six of 16 patterns adapted from Castilian and Andalusian traditional songs (accompanying and identity functions in origin); used here for socio-educational purposes.	Same domain and same music.
4 Sound sources	Flat claps (≈60% of playing time), thigh pats (25%), chest pats (10%), seated heel stamps (5%).	No body sound: the same arm and leg trajectories performed in the air, without contact.
6 Interlimb organisation	Weeks 1–4 bimanual symmetric; 5–8 alternating with midline crossing; 9–12 hands and feet independent (maximum two motor streams). Mirror demonstration throughout.	Identical organisation and progression; mirror demonstration.
8 Tempo	72 bpm (weeks 1–4), 84 bpm (5–8), 96 bpm (9–12); metronome embedded in recorded backing tracks.	Identical tracks and tempi.
9 Metric structure	4/4; two-bar cycles progressing to four-bar; eighth-note subdivision; syncopation from week 7; rests on beats 2 and 4 from week 9. Notated examples would be supplied as Supplementary material.	Same cycles, performed as silent movement.
12 Transmission	Mirror imitation plus rhythmic syllables; no notation.	Same.
13 Provenance	Sources of the six traditional patterns identified; ten patterns composed for the study.	Same music.
14 Concurrent tasks	Weeks 1–3: none (guided execution, then without model). Weeks 4–12: category naming (verbal), counting backwards by twos (numerical), stop on a coloured card (inhibition), change pattern on a whistle (switching); level recorded per activity on a six-level ordinal scale.	None; guided execution throughout.
15 Priority	“Keep both going; if you have to drop one, keep the rhythm.”	Not applicable.
18 Coordination	Circle of 12; unison (weeks 1–6), call and response (7–9), two interlocking parts (10–12).	Same formation and coordination demands.
19 Contact	Paired hand contact in 2 of 16 patterns from week 7; three steps (none, fingertip, palm); opt-out signal and non-contact alternative provided.	Same patterns and procedures.
29 Equivalence audit	36 sessions of 45 min (3 per week, 10:00–10:45); the same two instructors rotated across conditions; verbatim presentation scripts (to be supplied as Supplementary material), both introducing “a rhythm-and-movement programme for healthy ageing”; weekly ratings of enjoyment (0–10) and exertion (Borg scale).	Matched on every dimension; equivalence on enjoyment and exertion tested and reported.
32 Process measures	Accuracy and mean absolute asynchrony (ms) from a ceiling microphone in weeks 1, 6 and 12; group synchrony index; bidirectional dual-task cost at the inhibition, switching and heterogeneous levels.	Timing and group synchrony from wrist-worn accelerometers in the same weeks.

The example is constructed for illustration and does not describe a published study. The comparator removes two components (body sound and concurrent load); a design aiming to separate them would add a dismantling arm with body sound but without concurrent tasks.

## 6 Illustrative application to published reports

To examine whether the items can be completed from published reports, and where current reporting falls short, BODYPERC was applied to three reports. Because the purpose was illustrative, the reports were selected purposively rather than sampled, and the selection criteria are stated here so that the choice can be judged. Three criteria were applied. First, each report had to describe, in a peer-reviewed full-text publication, an intervention in which sound produced by the participants’ own bodies was a deliberate component. Second, the set had to span the range of designs and traditions the checklist is meant to accommodate: quantitative and qualitative designs, two-arm and multi-arm comparisons, and more than one pedagogical tradition. Third, the set had to include reports from the authors’ own research group, because a checklist proposed by the developer of a method should be shown to expose gaps in that method’s own literature. The reports selected were a two-group quasi-experimental study of selective attention in primary school (Romero-Naranjo et al., 2023) and a four-arm quasi-experimental study of executive functions and anxiety (Romero-Naranjo and Navarro-Camacho, 2026b), both within the BAPNE method, and a phenomenological study of primary-school teachers trained in the KeKeÇa body music approach (Bulut et al., 2025), included because it represents a different tradition and a qualitative design, the case for which the applicability of the checklist was least certain. Each item was rated as reported, partially reported, not reported or not applicable. For the qualitative study, items were rated for the teacher-training programme, which was the intervention its participants received. The ratings were made by a single rater, and their reliability has not been tested. Because the reports were chosen to span designs and traditions rather than to represent the literature, and two of them come from the authors’ group, the results below describe these three reports only: they show that the items can be completed and where these reports fall short, not how often the same gaps occur elsewhere. Whether the gaps are characteristic of the field is an empirical question for a structured reporting-quality audit. Its protocol, which specifies search strategies for five databases, eligibility criteria, a rule for selecting reports, independent double coding and the agreement statistics to be reported, is provided as Supplementary material 1, and the audit is the first of the next steps described in Section 7.5.

Figure 3 summarises the ratings. Of the 33 items, the three reports fully reported five, four and eight items respectively, and partially reported a further eleven, seventeen and seven. Only two items were fully reported in all three: the domain of application (item 3) and the use of voice (item 10). Nine items were reported in none of the three reports to which they applied: tempo (item 8), auditory context (item 11), priority instruction (item 15), independent practice (item 23), planned tailoring (item 26), unplanned modifications (item 27), the fidelity plan (item 30), in-session process measures (item 32) and adverse events (item 33).

**Figure 3:**
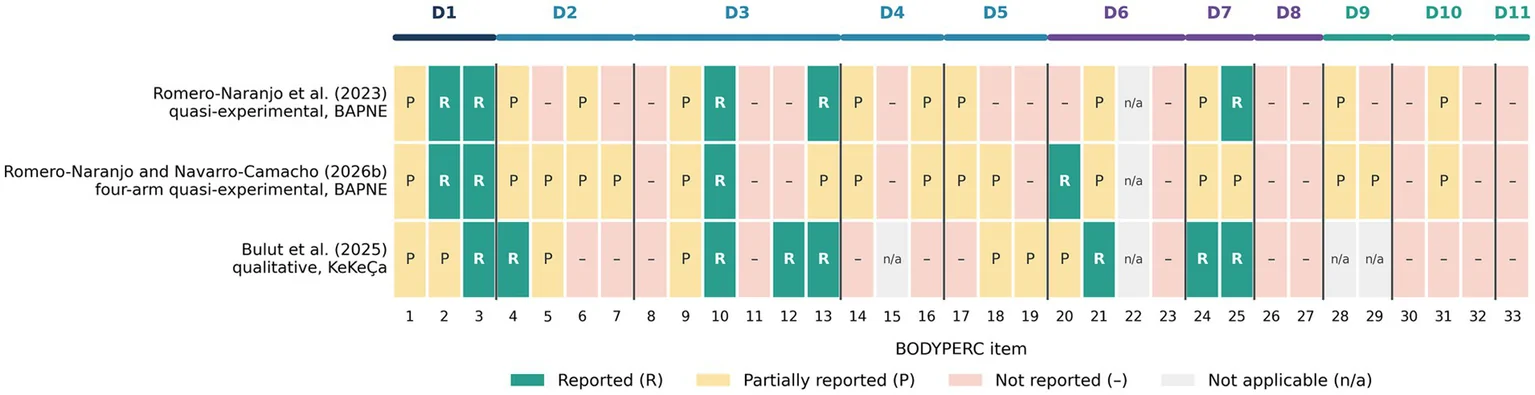
Illustrative application of BODYPERC to three published reports. Columns are the 33 items grouped by domain (D1–D11); rows are the reports. Cells show whether each item was reported, partially reported, not reported or not applicable. For Bulut et al. (2025), a qualitative study of teachers, items were rated for the teacher-training programme received by the study participants. Reports were selected purposively to span designs and traditions (see text), and two are by the authors’ research group. Ratings were made by a single rater, describe these three reports only and are offered as an illustration of the checklist’s use, not as an appraisal of study quality or an estimate of reporting quality in the literature.

Within these three reports, two patterns stand out. First, the gaps fall into two groups: features that generic templates do not prompt, such as tempo and the priority between rhythm and concurrent task, and features that existing guidelines do prompt but that were omitted nonetheless, such as auditory context, the fidelity plan and adverse events. Second, each report documents well some domain that another omits. The four-arm study provides a session structure with planned minutes for each module; the selective-attention study specifies its assessor and its attendance threshold; and the qualitative study describes its sound vocabulary, transmission mode and repertoire sources in detail. That each report supplies part of what another lacks is consistent with the interpretation that the missing items reflect the absence of a shared template rather than a lack of information available to the authors, which is the problem a reporting guideline is designed to solve.

## 7 Discussion

### 7.1 Relationship to existing guidelines

BODYPERC is intended to be used together with, not instead of, existing guidelines. For a randomised trial of a body percussion intervention, CONSORT (or CONSORT-SPI) governs the trial report, TIDieR governs the intervention description, CERT applies where physical-activity outcomes or exercise dose are central, and BODYPERC adds the features that make the intervention a body percussion intervention. For quasi-experimental studies, which make up much of the body percussion literature, TREND plays the role of CONSORT (Des Jarlais et al., 2004). For protocols, BODYPERC items can be addressed within the intervention and comparator sections required by SPIRIT (Chan et al., 2025). This layered design follows the precedent of the TIDieR extensions for population health and for placebo controls (Campbell et al., 2018; Howick et al., 2020). BODYPERC is not restricted to trials, because qualitative and observational studies also depend on a clear account of what participants experienced. A phenomenological study of teachers using body music games, for example, documented the teacher-training programme in detail, as its design required, and listed the games used, but did not report at what tempo or for how long pupils played them (Bulut et al., 2025); the items of domains 2 to 6 would allow such studies to document the intervention without changing their design. Most items concern information that investigators already hold, and the full checklist can be supplied as Table 3 when word limits are tight.

The music-intervention guidelines (Robb et al., 2011, 2025) are the closest precedent, and BODYPERC shares their conviction that the music itself must be described. It differs in treating the body as the instrument. This shifts attention from music selection to rhythm generation, from listening to motor execution, and from the interventionist’s musical choices to the participants’ interlimb organisation, cognitive load and coordination with one another. Dance interventions, which share many of these features, may in turn benefit from items developed here, particularly items 6, 8, 9, 14 and 18. Conversely, a future dance-specific extension could supply items on choreographic structure and spatial pathways that BODYPERC does not address.

### 7.2 Implications for movement science and clinical translation

Dance and body percussion interventions are increasingly expected to show through which motor-control, biomechanical and physiological processes they act, in addition to whether they work. Several BODYPERC items correspond to the mechanistic parameters of a rhythmic motor intervention: the effectors and body surfaces used (item 4), interlimb organisation (item 6), posture and locomotion (item 7), tempo and rhythmic structure (items 8 and 9), concurrent cognitive load (item 14) and physical intensity (item 22). When these parameters are reported consistently, outcomes can be related across studies to dose, coordination demand and cognitive load, for example through meta-regression, rather than to programme labels. This matters most for clinical translation. In Parkinson’s disease, rhythm-based rehabilitation has manipulated the rate of rhythmic cueing (Thaut et al., 1996) and the addition of a concurrent task (Park and Kim, 2021), and a clinician who wishes to apply a body percussion programme needs to know the tempo, the effectors and the load at which it was delivered. The process measures of item 32 add a further link between mechanism and outcome: timing precision, interpersonal synchrony and dual-task cost can be recorded with inexpensive microphones or wearable sensors during ordinary sessions and tested as mediators of clinical and educational effects. The same logic extends to biomechanical and physiological monitoring. Inertial sensors, pressure insoles and markerless motion capture allow joint excursion, impact loading, postural sway and heart-rate response to be recorded in community and clinical settings, but such data can be interpreted only if the motor task is known. A seated thigh-patting pattern and a standing stamping pattern at the same tempo differ in balance demand and impact load, and items 4, 6, 7 and 22 supply this context.

### 7.3 Standardisation, adaptation and cultural provenance

A reporting guideline is not a prescription, and BODYPERC does not ask any intervention to be standardised; it asks that whatever was done be described. This distinction matters for a practice rooted in oral traditions, where variation is the norm and where insisting on uniform form could erase what gives a practice its meaning. Item 26 applies the function–form distinction (Hawe et al., 2004) to this problem: investigators can hold constant what they hypothesise to be active, such as the load level or the coordination demand, while allowing repertoire, language and style to reflect local practice, and report both. Items 3 and 13 add an obligation that is ethical as well as scientific: when interventions borrow from living traditions, reports should say so. This also makes it possible to study whether the cultural meaning of a practice contributes to its effects.

### 7.4 Limitations

The main limitation of BODYPERC is that it has not yet been through a consensus process. Its items were derived by two authors from the same institution through conceptual analysis of existing guidelines and literature, and the illustrative application in section 6 used a single rater and three purposively selected reports, two of them from the authors’ group, so it shows that the items can be completed but not how often the gaps it identified occur in the wider literature. The classification of items as new, extended or adopted, and as core or contextual, reflects judgements that others may not share. The authors’ involvement in a named body percussion method, as its developer and as teachers, is a potential source of bias despite the safeguards described in section 3, which is a further reason for the consensus stage to be led by an independent panel. Several sources that elaborate specific constructs, such as the ordinal load scale, the comparator taxonomy and the contact procedures, are by the first author and are published in Spanish; each is cited alongside the international literature on which it draws, and no item depends on accepting any of them. Second, the checklist has not been tested for usability or for the reliability with which different raters extract its items from published reports. Third, some items rest on provisional evidence, for example the hypothesis that effector mechanics constrain preferred tempo, or the proposed ordinal scale of cognitive load. BODYPERC asks for these features to be reported because their importance is uncertain, and consistent reporting is the precondition for testing them. Fourth, the boundaries of the intervention class are not sharp, and terminology varies: some traditions prefer body music, a broader term that includes vocal and mouth sounds (Bulut et al., 2025). Body percussion shades into dance, drumming and vocal music, and the checklist will need explicit guidance on when it applies. A provisional criterion is that it applies whenever sound production by the participants’ own bodies is a deliberate component of the intervention.

### 7.5 Next, steps

Following the sequence recommended by Moher et al. (2010), four further steps are required before BODYPERC can be recommended as a standard. The first is a reporting-quality audit that applies the checklist, following the protocol in Supplementary material 1 and with independent double coding, to the controlled studies of body percussion interventions published to date. It will establish a baseline, show whether the gaps observed in Section 6 are characteristic of the literature, and identify items that are unworkable in practice. The second is an international modified Delphi study reported according to CREDES (Jünger et al., 2017), with a panel spanning music education, dance, music therapy, rehabilitation, ethnomusicology and research methodology and including practitioners of several body percussion traditions and methods, so that no single method is over-represented. The third is an inter-rater reliability study of item extraction from published reports. The fourth is an explanation-and-elaboration document with examples of good reporting for each item, together with registration of the guideline as under development with the EQUATOR Network. Researchers and practitioners are invited to use the present version in protocols and reviews and to report the difficulties they encounter, since this feedback will inform the consensus process.

## 8 Conclusion

The evidence base for body percussion cannot be synthesised as long as studies are described by the name of a programme rather than by its content. BODYPERC proposes 33 items that make the content of body percussion interventions and their comparators visible: what the body plays, at what tempo and with what structure, under what cognitive load, with whom and in what formation, how much, against what, and how well. Because the intervention produces sound, its delivery can be verified from recordings of the sessions, an opportunity that few other interventions offer. The checklist is method-neutral by design and provisional by necessity. Its value will depend on whether researchers from many traditions and methods are willing to test it, criticise it and turn it into a shared standard.
