# Supplementary material for: Describing what the body plays: the BODYPERC checklist for reporting body percussion interventions, a method-neutral extension of TIDieR and CERT
Source: Front Psychol. 2026 Oct 22;17:2004833. doi: 10.3389/fpsyg.2026.2004833 (PMC13735545; doi:10.3389/fpsyg.2026.2004833)
Supplement: Supplementary file 1 [file Supplementary_file_1.docx]

**Supplementary Material 1**

**Protocol for a reporting-quality audit of body percussion intervention studies using BODYPERC**

This protocol accompanies the article “Describing what the body plays: the BODYPERC checklist for reporting body percussion interventions, a method-neutral extension of TIDieR and CERT”. It specifies the structured, reproducible procedure by which the gaps identified in three purposively selected reports (Section 6 of the article) will be tested against the wider literature. It will be registered on the Open Science Framework before screening begins, and any deviation will be reported.

## 1. Objectives

(a) To estimate, for each of the 33 BODYPERC items, the proportion of controlled studies of body percussion interventions that report the item fully, partially or not at all. (b) To establish whether the gaps observed in the illustrative application (tempo, auditory context, priority instruction, independent practice, planned tailoring, unplanned modifications, fidelity plan, process measures and adverse events) are characteristic of the literature. (c) To identify items that raters cannot apply reliably, as input to the Delphi stage. (d) To estimate inter-rater agreement for item extraction.

## 2. Eligibility criteria

| **Criterion** | **Definition** |
| --- | --- |
| Intervention | Any intervention in which sound produced by the participants’ own bodies (clapping, snapping, patting, stamping, stepping, body music, vocal percussion combined with body sounds) is a deliberate and described component, whatever the method, tradition or label. Multi-component programmes are eligible if body percussion is named as a component. |
| Comparator | At least one comparison condition: randomised, cluster-randomised or quasi-experimental controlled designs, including multi-arm designs and controlled within-participant phase designs. Single-group pre–post studies are recorded but analysed separately. |
| Participants and setting | Any age, population and setting (educational, clinical, rehabilitative, community). |
| Outcomes | Any outcome. Outcomes are not used to select studies. |
| Publication | Peer-reviewed journal articles with full text available; 2001 to the date of the search (aligned with the bibliometric survey by Arnau-Mollá and Romero-Naranjo, 2024). Languages: English, Spanish, Portuguese, Italian, French and Turkish; other languages if a translation can be obtained. |
| Exclusions | Conceptual, descriptive or bibliometric papers without an intervention; studies in which clapping or tapping is only an outcome measure, a test or a clinical sign; performance reports; conference abstracts; theses (recorded for sensitivity analysis only); non-human studies. |

## 3. Information sources

Scopus, Web of Science Core Collection, PubMed/MEDLINE, ERIC and Dialnet. Five databases are required because much of this literature is published in education and sport-science journals in Spanish and Portuguese that are not indexed in biomedical databases. Supplementary sources: the reference lists of included studies, forward citation searching of included studies in Google Scholar, and the corpus of the bibliometric survey by Arnau-Mollá and Romero-Naranjo (2024), which will be cross-checked against the database results.

## 4. Search strategies

The strategies combine terms for the practice with terms for an intervention. They will be piloted, and the final strings, the date of each search and the number of records retrieved from each source will be reported.

*Scopus*

TITLE-ABS-KEY ( "body percussion" OR "body music" OR "body drumming" OR "percusión corporal" OR "percussão corporal" OR "percussione corporea" OR "percussion corporelle" OR "beden perküsyonu" OR "hand-clapping game*" OR "handclapping game*" OR "clapping game*" OR "clapping song*" OR "gumboot danc*" OR "hambone" OR "BAPNE" OR "KeKeÇa" OR "neuromotricity" OR "neuromotricidad" ) AND TITLE-ABS-KEY ( intervention* OR program* OR training OR trial OR "quasi-experiment*" OR experiment* OR effect* OR teaching OR therap* OR rehabilitat* ) AND PUBYEAR > 2000

*Web of Science Core Collection*

TS=("body percussion" OR "body music" OR "body drumming" OR "percusión corporal" OR "percussão corporal" OR "percussione corporea" OR "percussion corporelle" OR "beden perküsyonu" OR "hand-clapping game*" OR "handclapping game*" OR "clapping game*" OR "clapping song*" OR "gumboot danc*" OR "hambone" OR "BAPNE" OR "KeKeÇa" OR "neuromotricity" OR "neuromotricidad") AND TS=(intervention* OR program* OR training OR trial OR "quasi-experiment*" OR experiment* OR effect* OR teaching OR therap* OR rehabilitat*) [Timespan: 2001-01-01 to date of search]

*PubMed/MEDLINE*

("body percussion"[tiab] OR "body music"[tiab] OR "body drumming"[tiab] OR "percusión corporal"[tiab] OR "hand-clapping game*"[tiab] OR "handclapping"[tiab] OR "clapping game*"[tiab] OR "clapping song*"[tiab] OR "BAPNE"[tiab] OR "neuromotricity"[tiab]) AND (intervention*[tiab] OR program*[tiab] OR training[tiab] OR trial[tiab] OR experiment*[tiab] OR effect*[tiab] OR teaching[tiab] OR therap*[tiab] OR rehabilitat*[tiab]) AND 2001:3000[dp]

*ERIC (EBSCOhost or ProQuest)*

AB,TI ( "body percussion" OR "body music" OR "body drumming" OR "clapping game*" OR "clapping song*" OR "hand-clapping" OR "handclapping" OR "BAPNE" OR "KeKeÇa" ) AND ( intervention* OR program* OR training OR experiment* OR effect* OR teaching )

*Dialnet*

"percusión corporal" OR "body percussion" OR "percussão corporal" OR "BAPNE" OR "neuromotricidad" [Documento: artículo de revista; búsqueda en título, resumen y palabras clave]

## 5. Study selection

Records will be deduplicated in a reference manager. Two reviewers will independently screen titles and abstracts and then full texts against the criteria in section 2, recording a reason for each full-text exclusion. At least one reviewer at each stage will be independent of the BAPNE research group. Disagreements will be resolved by discussion and, if needed, by a third reviewer. The process will be reported with a PRISMA 2020 flow diagram (Page et al., 2021).

## 6. Sampling rule

All eligible controlled studies will be coded. If more than 80 are eligible, a simple random sample of 80 will be drawn, stratified by research-group affiliation (studies by the BAPNE research group versus all others) in proportion to their share of the eligible set, using a random seed that will be reported. Because a single research group has produced a large share of this literature (Arnau-Mollá and Romero-Naranjo, 2024), all results will also be reported separately for the two strata, so that conclusions about the field are not driven by one group’s reporting practices.

## 7. Data extraction and coding

Each of the 33 BODYPERC items will be rated as reported, partially reported, not reported or not applicable, for the intervention and, where applicable, for each comparator (item 28). Each rating will be supported by a verbatim quotation with its page number, or by a note that no relevant text was found. Supplementary materials, protocols and registrations cited in the article will be consulted. A coding manual will define the decision rule for each item before coding begins; for example, item 8 (tempo) is rated as reported when a numerical tempo, inter-onset interval or metronome setting is given, as partially reported when tempo is described only qualitatively (for example, “slow”), and as not reported otherwise. The manual will be calibrated on three eligible studies that are then excluded from the agreement analysis, and revised once before formal coding.

Two raters will code every study independently. At least one rater will be independent of the authors’ research group, and studies co-authored by a rater will be coded by another rater. Descriptive data will also be extracted: year, country, language, design, population, setting, named method or tradition, number of arms and research-group affiliation.

## 8. Agreement

Pre-consensus agreement will be reported for each item as percentage agreement, Cohen’s kappa (Cohen, 1960) and Gwet’s AC1 (Gwet, 2008), which is less affected by the skewed prevalence expected for rarely reported items, each with a 95% confidence interval. Disagreements will then be resolved by discussion, and the consensus ratings will be used in the analysis. Items with AC1 below 0.60 will be flagged for revision at the Delphi stage.

## 9. Analysis

For each item and domain, the proportion of studies reporting the item fully and at least partially will be reported with Wilson 95% confidence intervals (Wilson, 1927), computed over the studies to which the item applies. Results will be described by research-group affiliation, design and period of publication (before and after the publication of TIDieR in 2014). The analysis is descriptive; no study-level quality score will be computed, because BODYPERC is a reporting guideline and not a tool for appraising risk of bias.

## References

Arnau-Mollá, A. F., and Romero-Naranjo, F. J. (2024). Evolución bibliométrica de la percusión corporal: impacto y género en las publicaciones científico-académicas. Retos 51, 1025–1054. doi: 10.47197/retos.v51.101450

Cohen, J. (1960). A coefficient of agreement for nominal scales. Educ. Psychol. Meas. 20, 37–46. doi: 10.1177/001316446002000104

Gwet, K. L. (2008). Computing inter-rater reliability and its variance in the presence of high agreement. Br. J. Math. Stat. Psychol. 61, 29–48. doi: 10.1348/000711006X126600

Page, M. J., McKenzie, J. E., Bossuyt, P. M., Boutron, I., Hoffmann, T. C., Mulrow, C. D., et al. (2021). The PRISMA 2020 statement: an updated guideline for reporting systematic reviews. BMJ 372:n71. doi: 10.1136/bmj.n71

Wilson, E. B. (1927). Probable inference, the law of succession, and statistical inference. J. Am. Stat. Assoc. 22, 209–212. doi: 10.1080/01621459.1927.10502953
